# Medical Student Musculoskeletal Knowledge: Examining the Impact and Value of an Orthopaedic Surgery Clerkship Using the Freedman and Bernstein Examination

**DOI:** 10.1186/s12909-025-06673-2

**Published:** 2025-01-29

**Authors:** Nicole Nishime, Mary Seibel, Dieter Lindskog, Daniel Wiznia

**Affiliations:** 1 Frank H. Netter MD School of Medicine at Quinnipiac University, 370 Bassett Rd, North Haven, CT 06473 USA; 2 Department of Orthopaedics and Rehabilitation, Yale School of Medicine, 47 College Street, 2nd Floor, New Haven, CT 06510 USA

**Keywords:** Medical education, Medical students, Musculoskeletal, Freedman–Bernstein

## Abstract

**Purpose:**

Given the importance of musculoskeletal knowledge but the limited orthopaedic instruction offered in medical school, our Orthopaedic Surgery Department developed a three-week clerkship for interested students. This study assesses the clerkship’s impact on medical student musculoskeletal knowledge through administration of the Freedman and Bernstein Basic Cognitive Musculoskeletal Examination.

**Methods:**

Medical students enrolled in the orthopaedic surgery clerkship between February 2019 and May 2024 were asked to participate in pre- and post-clerkship surveys using the Freedman and Bernstein Basic Cognitive Musculoskeletal Examination. Raw and weighted scores were computed according to the guidelines provided by Freedman and Bernstein. Averaged scores were used to compute mean pre- and post-test scores.

**Results:**

There were 64 responses to the pre-test and 33 responses to the post-test. The mean pre-test weighted score was 54% with 12 students (18.8%) passing. The mean post-test score was 70% with 17 students (51.5%) passing. Raw scores showed that musculoskeletal knowledge improved from pre-test (M = 55.13, SD = 19.90) to post-test (M = 70.22, SD = 14.70; *p* < .001). The results comparing weighted scores showed that the participants’ musculoskeletal knowledge also improved from pre-test (M = 52.86, SD = 21.12) to post-test (M = 67.11, SD = 19.02; *p* < .001).

**Conclusion:**

While students demonstrated improved musculoskeletal knowledge after completing our institution’s orthopaedic surgery clerkship, almost half of the students did not pass the post-test. Most of the students who did not pass the post-test expressed definite or possible interest in pursuing an orthopaedic surgery residency.

## Background

Musculoskeletal disorders are the leading cause of disability worldwide, affecting approximately one in two U.S. adults [1, 2]. While musculoskeletal complaints account for approximately 20% of all visits to primary care clinics and emergency departments, the average time spent on musculoskeletal instruction in medical schools is 2 to 3 weeks [3, 4]. Even though knowledge of musculoskeletal disorders is important for a physician in any specialty, musculoskeletal clinical instruction is not considered a traditional medical school core clerkship and is often only offered as an optional selective [5, 6].

To test fundamental musculoskeletal knowledge, Freedman and Bernstein developed a basic competency exam [3]. The exam was validated by both orthopaedic and internal medicine residency program directors in the United States; they were asked to rate the importance of each question and suggest a passing score. The exam was then administered to first-year surgical and internal medicine residents at the authors’ institution on their first day of residency. Since then, this exam has been widely distributed and it has been demonstrated that the majority of individuals at multiple levels of medical training (medical students, recent medical school graduates, and non-orthopaedic residents) are unable to achieve a passing score on this exam [3, 4, 7–11]. In a study conducted by the University of Washington Medical School, senior medical students who had taken an optional clinical musculoskeletal elective scored higher on a modified Freedman and Bernstein exam compared to students who had not completed the elective [9].

When analyzing the impact of curricular development, medical school instructors have found pre- and post-tests are useful to evaluate the direct impact of their teaching sessions and modules [12, 13]. While there is limited data utilizing the Freedman and Bernstein exam as a pre- and post-test, one study used this exam to demonstrate significant improvement in musculoskeletal knowledge among emergency medicine residents after completing either a traditional orthopaedic surgery rotation or a primary care sports medicine rotation [14]. Since the creation of the Freedman and Bernstein exam, a new exam has been developed and validated to assess medical student musculoskeletal knowledge specifically; however, this tool cannot be administered to the same individual twice due to the multiple-choice nature of the exam [15].

As the inadequacy of medical student musculoskeletal knowledge is becoming more widely recognized, medical schools have begun to design and implement modules aimed to better prepare students [16, 17]. Over the last five years, our institution has instituted an optional three-week orthopaedic clerkship embedded within a 12-week second- and third-year medical student general surgery clerkship. Students are assigned to inpatient or outpatient subspecialty teams and are active participants in the resident education program. While the overarching general surgery clerkship includes classes specifically for medical students, the orthopaedic clerkship itself is missing this component. Instead, students attend weekly education sessions within the assigned subspecialty as well as spend one half day a week in the resident education program. The purpose of this study was to evaluate if our three-week orthopaedic clerkship improves medical student musculoskeletal knowledge by comparing pre- and post-clerkship scores on the Freedman and Bernstein exam.

## Methods

Medical students enrolled in our institution’s Orthopaedic Surgery Clerkship between February 2019 and May 2024 were asked to volunteer to participate in this quality improvement initiative. The optional three-week musculoskeletal clerkship includes both inpatient and outpatient orthopaedic rotations that second- and third-year medical students can take as part of their general surgery rotation. Subspecialty services within this clerkship included orthopaedic trauma and fracture care, adult reconstruction, orthopaedic oncology, sports medicine, spine, hand and upper extremity, and foot and ankle. As the study involved a quality improvement project to evaluate the educational impact of the clerkship, institutional review board approval was not required. All students were informed that completing both pre- and post-clerkship surveys was voluntary and would not affect their surgery rotation evaluations.

Students received links to pre- and post-clerkship surveys. These surveys assessed fundamental knowledge of musculoskeletal topics before and after completing the clerkship using the validated Freedman and Bernstein Basic Cognitive Musculoskeletal Examination. Additional information collected included participants’ interest in applying to orthopaedic surgery residency and plans to take a research year. The post-clerkship survey also assessed the amount of time spent on each service during the three-week rotation. The students were asked to take these tests untimed, closed book, and at their convenience.

Each participant was assigned a unique identifier to allow for comparison between pre- and post-test scores. All questionnaires were blinded prior to grading, with raw and weighted scores computed according to the guidelines provided by Freedman and Bernstein and the recommended passing score set at 73.1%. Each test was scored independently by two raters who were blind to the other rater’s scores. Inter-rater reliability was assessed using an intraclass correlation coefficient (ICC). The averaged weighted scores were then used to compute mean pre- and post-test raw scores. Score improvement between pre- and post-tests was evaluated using a paired samples t-test.

## Results

There were 64 responses to the pre-test and 33 responses to the post-test (51.6% retention rate). For the pre-test, the majority of respondents were second-year medical students (54.7%), followed by third-year (37.5%), fourth-year (6.3%), and one first-year student (1.6%) (Table 1). For the post-test, respondents were second- (54.5%), third- (42.4%), or fourth-year (3.0%) medical students (Table 2). Among the students who completed the pre-test, 50.0% were interested in applying to an orthopaedic surgery residency, compared to 63.6% who completed the post-test. Most students spent at least a portion of their elective on the trauma service (72.7%), followed by total joint (39.4%), and sports medicine (21.2%) (Table 3).

**Table 1:** Pre-Test Student Demographics (*n* = 64)

Year in Medical School, *n* (%)	
MS 1	1 (1.6)
MS 2	35 (54.7)
MS 3	24 (37.5)
MS 4	4 (6.3)
Interest in applying to orthopaedic surgery residency, n (%)	
Yes	32 (50.0)
Maybe	24 (37.5)
No	8 (12.5)
Plans to take a research year, n (%)	
Yes	33 (51.6)
Maybe	23 (35.9)
No	8 (12.5)

**Table 2:** Post-Test Student Demographics (*n* = 33)

Year in Medical School, *n* (%)	
MS 1	0 (0)
MS 2	18 (54.5)
MS 3	14 (42.4)
MS 4	1 (3.0)
Interest in applying to orthopaedic surgery residency, n (%)	
Yes	21 (63.6)
Maybe	10 (30.3)
No	2 (6.1)
Plans to take a research year, n (%)	
Yes	18 (54.5)
Maybe	12 (36.4)
No	3 (9.1)

**Table 3:** Total Time Spent on Each Subspecialty (*n* = 33)

	Total Joint	Hand	Spine	Trauma	Sports	Foot and Ankle	Musculoskeletal Oncology
None	20 (60.6)	31 (93.9)	33 (100)	9 (27.3)	26 (78.8)	33 (100)	30 (90.9)
Less than 1 week	1 (3.0)	2 (6.1)	0 (0)	2 (6.1)	2 (6.1)	0 (0)	2 (6.1)
2 weeks	8 (24.2)	0 (0)	0 (0)	10 (30.3)	0 (0)	0 (0)	0 (0)
3 weeks	4 (12.1)	0 (0)	0 (0)	12 (36.4)	5 (15.2)	0 (0)	1 (3.0)

The inter-rater reliability of the Freedman and Bernstein Basic Cognitive Musculoskeletal Examination was assessed using an intraclass correlation coefficient (ICC). For the raw scores, ICC single measures values equaled 0.982 (pre-test) and 0.943 (post-test). The weighted scores were then calculated according to the guidelines provided by Freedman and Bernstein. ICC single measures values for weighted scores equaled 0.984 (pre-test) and 0.966 (post-test). The weighted scores assigned by two independent raters were averaged to validate the reliability of the musculoskeletal examination. The mean pre-test weighted score was 54% with only 12 students (18.8%) passing. The mean post-test score was 70%, with 17 students (51.5%) passing. Among the students who did not pass the post-test, 7 (43.8%) expressed interest in applying to orthopaedic surgery residency, 8 (50.0%) expressed possible interest, and 1 (6.3%) expressed no interest.

**Fig. 1:**
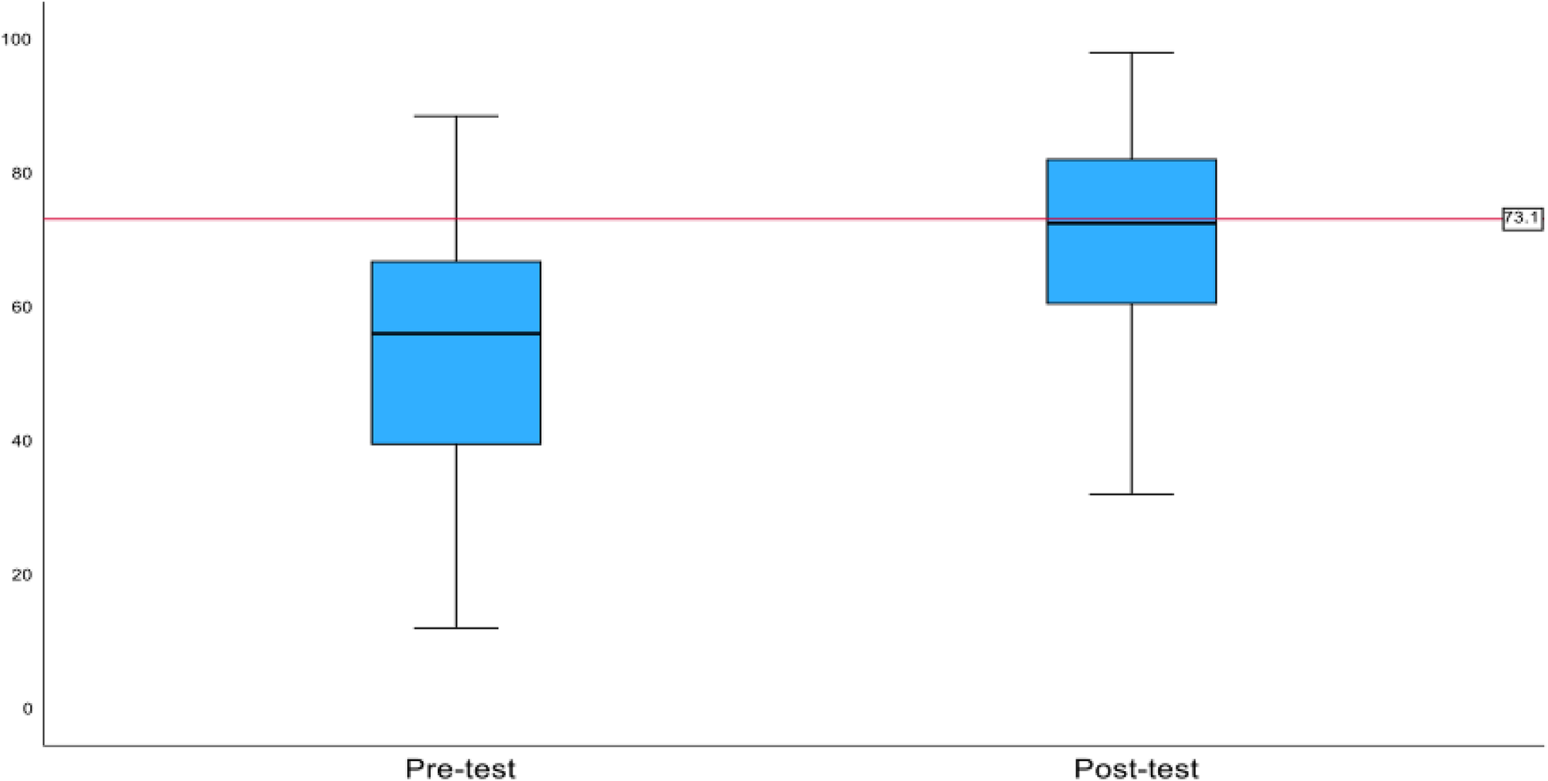
Average Raw Pre-Test and Post-Test Scores

**Fig. 2:**
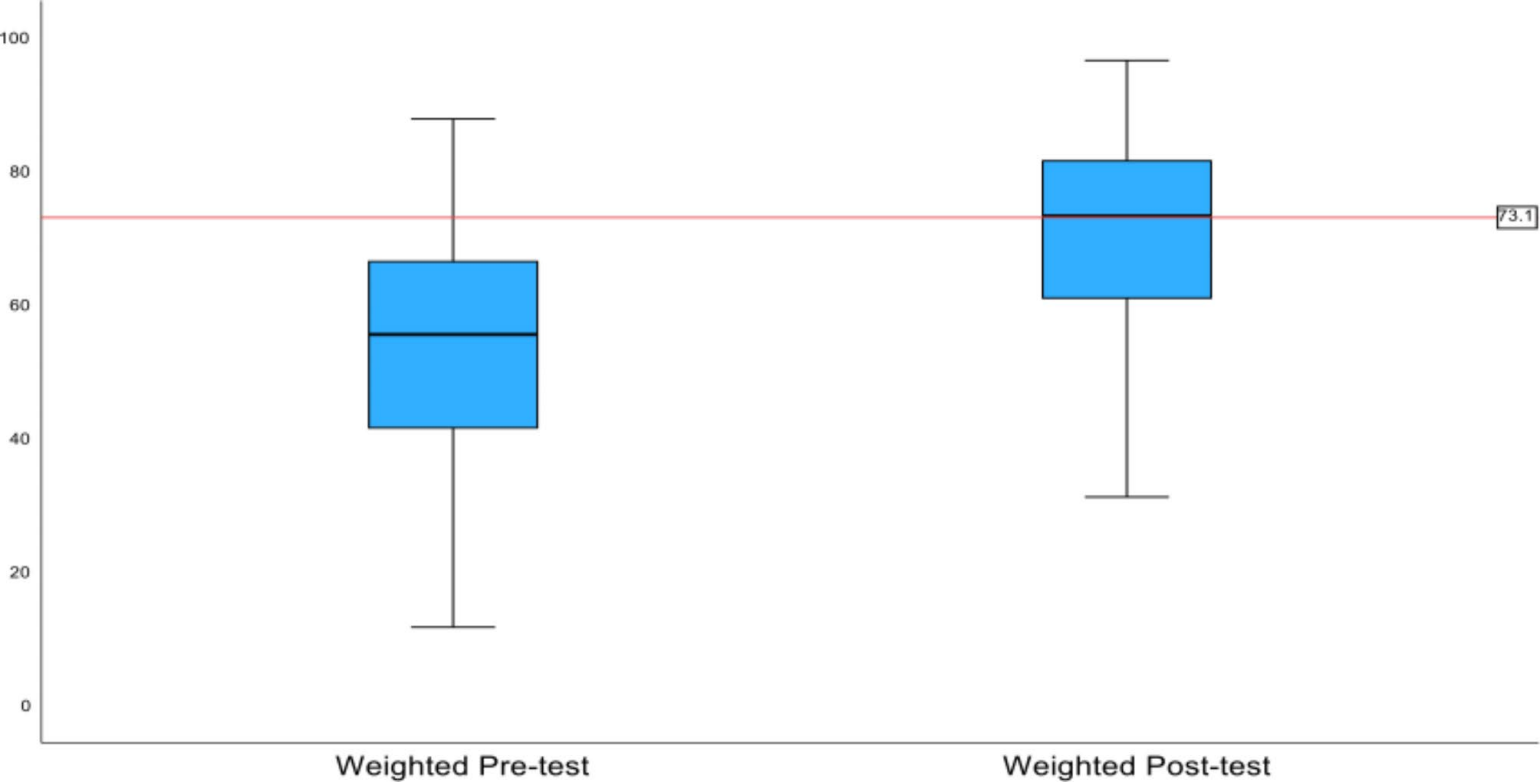
Average Weighted Pre-Test and Post-Test Scores

A paired t-test analysis was conducted to compare the raw and weighted pre-test and post-test scores of the participants. Raw scores showed that musculoskeletal knowledge improved from pre-test (M = 55.13, SD = 19.90, 95% CI = [50.25, 60.01]) to post-test (M = 70.22, SD = 14.70; *p* < .001, 95% CI = [65.01, 75.43]) (Fig. 1). The results comparing weighted scores showed that the participants’ musculoskeletal knowledge also improved from pre-test (M = 52.86, SD = 21.12, 95% CI = [47.69, 58.03]) to post-test (M = 67.11, SD = 19.02; *p* < .001, 95% CI = [60.62, 73.60]) (Fig. 2).

Raw scores showed that musculoskeletal knowledge improved from pre-test (M = 55.13, SD = 19.90, 95% CI = [50.25, 60.01]) to post-test (M = 70.22, SD = 14.70; *p* < .001, 95% CI = [65.01, 75.43]).

The results comparing weighted scores showed that the participants’ musculoskeletal knowledge also improved from pre-test (M = 52.86, SD = 21.12, 95% CI = [47.69, 58.03]) to post-test (M = 67.11, SD = 19.02; *p* < .001, 95% CI = [60.62, 73.60]).

When analyzing the percentage correct for individual pre- and post-test questions, percent correct was determined from an average of both raters’ scores. Utilizing this average of percent correct per question, less than 50% of students answered post-test questions correctly on the following topics: orthopaedic trauma, spine surgery, foot and ankle surgery, and musculoskeletal oncology (i.e. exam questions pertaining to treatment of open fractures and displaced fractures of the femoral neck, differential diagnoses for low back pain that wakes someone up at night and injuries that cause ankle soft tissue swelling, indications for ordering plain radiographs for new-onset back pain, and bone malignancies not detected by bone scans) (Table 4).

**Table 4:** Average Percentage Correct on Pre- and Post-Test Questions (*n* = 33)

Question	Topic/Relevant Subspecialty	Average % Correct on Pre-Test	Average % Correct on Post-Test
What common problem must all newborns be examined for?	Pediatric/Total Joint	71.09	87.88
What is a compartment syndrome?	Trauma	88.28	90.91
Acute septic arthritis of the knee may be differentiated from inflammatory arthritis by which laboratory test?	Total Joint	72.66	77.27
A patient dislocates his knee in a car accident. What structure(s) is/are at risk for injury and therefore must be evaluated?	Trauma	35.94	54.55
A patient punches his companion in the face and sustains a fracture of the 5th metacarpal and a 3-mm break in the skin over the fracture. What is the correct treatment, and why?	Trauma/Hand	31.64	34.09
A patient comes to the office complaining of low-back pain that wakes him up from sleep. What two diagnoses are you concerned about?	Spine/Musculoskeletal Oncology	24.61	34.85
How is compartment syndrome treated?	Trauma	87.50	98.49
A patient lands on his hand and is tender to palpation in the “snuff box” (the space between the thumb extensor and abductor tendons). Initial radiographs do not show a fracture. What diagnosis must be considered?	Trauma/Hand	56.25	81.82
A 25-year-old male is involved in a motor vehicle accident. His left limb is in a position of flexion at the knee and hip, with internal rotation and adduction of the hip. What is the most likely diagnosis?	Trauma	49.22	71.21
What nerve is compressed in carpal tunnel syndrome?	Hand/Total Joint	95.31	100.00
A patient has a disc herniation pressing on the 5th lumbar nerve root. How is motor function of the 5th lumbar nerve root tested?	Spine	17.97	50.00
How is motor function of the median nerve tested in the hand?	Hand/Total Joint	46.09	81.82
A 12-year-old boy severely twists his ankle. Radiographs show only soft-tissue swelling. He is tender at the distal aspect of the fibula. What are 2 possible diagnoses?	Trauma/Foot and Ankle/Total Joint	33.20	44.70
A patient presents with new-onset low back pain. Under what conditions are plain radiographs indicated? Please name 5 (example: history of trauma).	Spine/Musculoskeletal Oncology	20.31	29.17
A patient has a displaced fracture near the fibular neck. What structure is at risk for injury?	Trauma/Foot and Ankle	37.50	72.73
A 20-year-old injured his knee while playing football. You see him on the same day, and he has a knee effusion. An aspiration shows frank blood. What are the three most common diagnoses?	Trauma/Total Joint/Sports	49.61	75.76
What are the five most common sources of cancer metastatic to bone?	Musculoskeletal Oncology	56.45	79.92
Name two differences between rheumatoid arthritis and osteoarthrosis.	Total Joint	58.98	79.55
Which malignancy may be present in bone yet typically is not detected with a bone scan?	Musculoskeletal Oncology	28.13	24.24
What is the function of the normal anterior cruciate ligament at the knee?	Total Joint/Sports	67.97	89.39
What is the difference between osteoporosis and osteomalacia?	Total Joint	56.25	65.15
In elderly patients, displaced fractures of the femoral neck are typically treated with joint replacement, whereas fractures near the trochanter are treated with plates and screws. Why?	Total Joint/Trauma	41.41	45.46
What muscle(s) is/are involved in lateral epicondylitis (tennis elbow)?	Total Joint/Sports/Hand	29.69	74.24
Rupture of the biceps at the elbow results in weakness of both elbow flexion and ___?	Total Joint/Trauma/Sports/ Hand	53.91	87.88
What muscle(s) control(s) external rotation of the humerus with the arm at the side?	Total Joint/Sports/Hand	59.38	81.82

## Discussion

This study represents a novel approach in evaluating the teaching efficacy of an orthopaedic surgery clerkship by administering the full Freedman and Bernstein Basic Cognitive Musculoskeletal Examination as both a pre- and post-test. While previous studies have assessed musculoskeletal knowledge using this examination, none have utilized it to assess an orthopaedic surgery clerkship’s direct impact on medical students’ musculoskeletal knowledge. Our analysis demonstrated that students showed a significant increase from pre- to post-test scores, indicating that the small subset of students who submitted the post-test had improved musculoskeletal knowledge after completing this optional orthopaedic surgery clerkship. Despite the higher post-test scores, only slightly more than 50% of students passed the exam post rotation, including a majority of those expressing an interest in orthopedic surgery.

The assessment of inter-rater reliability demonstrated strong agreement between raters for both raw and weighted scores, indicating the consistency and reliability of the examination scoring process. These findings are particularly significant in the context of evaluating teaching efficacy, as they affirm the validity of the examination results in assessing student competency in musculoskeletal health following the clerkship.

The comparison of pre- and post-test scores revealed a significant improvement in musculoskeletal knowledge among the subset of participants who completed both assessments. Both raw and weighted scores showed substantial increases from the pre-test to the post-test, indicating the clerkship’s effectiveness in facilitating learning and knowledge retention. The higher percentage of students passing the post-test further underscores the positive impact of the clerkship on student musculoskeletal knowledge. However, the higher mean raw score as compared to the weighted score suggests that students were less knowledgeable on the questions that were deemed more important by the Freedman and Bernstein study [3].

When looking at individual exam questions, less than 50% of students were able to answer questions on certain high-yield and emergent topics, such as treatment and risks of an open fracture, even after completing the orthopaedic surgery clerkship. However, this may directly reflect the variable experiences within the clerkship; because students rotated on different subspecialties, individuals may have done worse on the topics they were not exposed to. As a result, the four main categories that students struggled with may be explained by the following: variable time spent on the trauma service, only a small subset with exposure to musculoskeletal oncology, and no exposure to the orthopaedic spine or foot and ankle services.

While the majority of students who passed the post-test were interested in pursuing orthopaedics, 93.8% of the students who did not pass also expressed definite or possible interest in applying to an orthopaedic residency. This suggests that lower scores were not a result of lack of interest in the specialty; however, it is also possible that students interested in orthopaedics were more likely to both choose to enroll in the optional clerkship and to later complete the post-test.

The study’s findings have important implications for orthopaedic surgery clerkship curriculum design and implementation. The demonstrated improvement in musculoskeletal knowledge suggests that the clerkship has the potential to enhance student learning outcomes in musculoskeletal health. Although there was a statistically significant improvement in musculoskeletal knowledge, the post-test results of only slightly more than 50% of students passing indicate that this may not be an accurate reflection of the effectiveness of the clerkship. These deficiencies suggest that educators can use these results to inform curriculum revisions and optimize teaching strategies to better prepare students for clinical practice when treating musculoskeletal complaints.

Our clerkship does not have a specific education program embedded in the rotation for medical students. Instead, students are taught alongside the residents, receiving the same lectures and proctoring. This suggests that there is an opportunity to provide education modules designed specifically for medical students, as the likely way medical students’ knowledge improved was due to clinical exposure or self-study. Because students rotate on different subspecialties within the clerkship, implementing classes with pre-selected common orthopaedic cases could ensure student exposure to each subspecialty to prevent any large gaps in knowledge.

## Limitations

The relatively small sample size and focus on a single institution’s orthopaedic surgery clerkship limit the generalizability of our findings. This highlights the need for larger-scale, multicenter studies to assess the impact of increased exposure to musculoskeletal conditions on medical students’ knowledge of these topics. Additionally, there were fewer post-test results as students were not required to complete the exam once they finished the orthopaedic clerkship. Many of the respondents to the post-test indicated that they are interested in pursuing an orthopaedic surgery residency, which may introduce both sampling and selection biases. Due to the low retention rate (51.6%) and possible biases, the results of this study may not accurately depict students’ change in musculoskeletal knowledge after completion of the clerkship.

The lack of standardization within the clerkship may have affected students’ performance on the exam as each received variable exposure to the offered subspecialties. Because the Freedman and Bernstein exam was validated amongst orthopaedic residency program chairpersons and originally administered to medical and surgical residents, the effectiveness of our clerkship may be hard to determine utilizing only this assessment. While other validated multiple-choice questionnaires have since been published that may provide helpful data regarding post-clerkship medical student musculoskeletal knowledge, the Freedman and Bernstein exam was chosen as it is widely used and validated in the United States. While the Freedman and Bernstein musculoskeletal questionnaire is highly reported in literature, the exam was first introduced and validated in 1998. Standards for assessing musculoskeletal knowledge have since evolved.

## Conclusion

This study provides valuable insights into the teaching efficacy of our orthopaedic surgery clerkship by administering the Freedman and Bernstein Basic Cognitive Musculoskeletal Examination as both a pre- and post-clerkship assessment. The change in scores demonstrated significantly improved knowledge among participants who completed the post-test, affirming the clerkship’s positive impact on students’ musculoskeletal knowledge. These findings emphasize the potential of an orthopaedic surgery clerkship to improve musculoskeletal knowledge through increased exposure to musculoskeletal topics in medical school.

Despite these findings, the results from our study could be strengthened in several ways. As many students struggled on topics that they did not receive exposure to, standardization within the rotation (i.e. subspecialties rotated on) may provide students with a wider breadth of musculoskeletal knowledge. This could be done in multiple ways, such as giving students more subspecialty exposure or implementing lectures or modules specifically designed for medical students within the orthopaedic clerkship to ensure that specific learning objectives are met.

## Data Availability

The datasets used and/or analyzed during the current study are available from the corresponding author on reasonable request.

## Abbreviations

ICC: Intraclass correlation coefficient

## References

[1] National Academies of Sciences, Engineering, and Medicine. Selected Health conditions and Likelihood of improvement with treatment. Wash (DC): Natl Academies Press (US). 2020. 10.17226/25662. https://www.ncbi.nlm.nih.gov/books/NBK559511. 32687289

[2] Musculoskeletal health. World Health Organization. https://www.who.int/news-room/fact-sheets/detail/musculoskeletal-conditions (2022). Accessed 24 May 2024.

[3] Freedman KB, Bernstein J. The adequacy of medical school education in musculoskeletal medicine. J Bone Joint Surg Am. 1998;80(10):1421–7. 10.2106/00004623-199810000-00003. 9801210 10.2106/00004623-199810000-00003

[4] Al-Nammari SS, Pengas I, Asopa V, Jawad A, Rafferty M, Ramachandran M. The inadequacy of musculoskeletal knowledge in graduating medical students in the United Kingdom. J Bone Joint Surg Am. 2015;97(7):e36. 10.2106/JBJS.N.00488. 25834088 10.2106/JBJS.N.00488

[5] Wang T, Xiong G, Lu L, Bernstein J, Ladd A. Musculoskeletal Education in Medical Schools: a survey in California and Review of Literature. Med Sci Educ. 2020;31(1):131–6. 10.1007/s40670-020-01144-3. 34457873 10.1007/s40670-020-01144-3 PMC8368391

[6] DiGiovanni BF, Sundem LT, Southgate RD, Lambert DR. Musculoskeletal Medicine is underrepresented in the American Medical School Clinical Curriculum. Clin Orthop. 2016;474(4):901–7. 10.1007/s11999-015-4511-7. 26282389 10.1007/s11999-015-4511-7 PMC4773350

[7] Day CS, Yeh AC, Franko O, Ramirez M, Krupat E. Musculoskeletal medicine: an assessment of the attitudes and knowledge of medical students at Harvard Medical School. Acad Med J Assoc Am Med Coll. 2007;82(5):452–7. 10.1097/ACM.0b013e31803ea860. 10.1097/ACM.0b013e31803ea860 17457065

[8] Matzkin E, Smith EL, Freccero D, Richardson AB. Adequacy of education in musculoskeletal medicine. J Bone Joint Surg Am. 2005;87(2):310–4. 10.2106/JBJS.D.01779. 15687152 10.2106/JBJS.D.01779

[9] Schmale GA. More evidence of educational inadequacies in musculoskeletal medicine. Clin Orthop. 2005;437251–9. 10.1097/01.blo.0000164497.51069.d9. 10.1097/01.blo.0000164497.51069.d9 16056057

[10] Weiss K, Curry E, Matzkin E. Assessment of medical school musculoskeletal education. Am J Orthop (Belle Mead NJ). 2015;44(3):E64-7. PMID: 25750952. 25750952

[11] Haywood BL, Porter SL, Grana WA. Assessment of musculoskeletal knowledge in primary care residents. Am J Orthop (Belle Mead NJ). 2006;35(6):273-5. PMID: 16841789. 16841789

[12] Blythe M, Quinn KR, Helmer SD, Smith JL. Development of a Near peer clinical anatomy Review Session during the surgery clerkship: pre- and Post-test results among Third Year Medical Students. Kans J Med. 2022;15:293–7. 10.17161/kjm.vol15.16372. 36042835 10.17161/kjm.vol15.16372 PMC9409865

[13] Wilder C, Kilgore LJ, Fritzel A, Larson KE. Improving Medical Student anatomy knowledge and confidence for the breast Surgical Oncology Rotation. Healthc Basel Switz. 2023;11(5):709. 10.3390/healthcare11050709. 10.3390/healthcare11050709 PMC10000369 36900714

[14] Denq W, Fox JD, Lane A, Caballero B, Godfrey B, Yim J, Hughes KE, Cahir TM, Waterbrook A. Impact of sports Medicine and orthopedic surgery rotations on Musculoskeletal Knowledge in Residency. Cureus. 2021;13(3):e14211. 10.7759/cureus.14211. PMID: 33948401; PMCID: PMC8086753. 33948401 10.7759/cureus.14211 PMC8086753

[15] Cummings DL, Smith M, Merrigan B, Leggit J. MSK30: a validated tool to assess clinical musculoskeletal knowledge. BMJ Open Sport Exerc Med. 2019;5(1):e000495. 10.1136/bmjsem-2018-000495. PMID: 30899552; PMCID: PMC6407547. 10.1136/bmjsem-2018-000495 PMC6407547 30899552

[16] Vioreanu MH, O’Daly BJ, Shelly MJ, Devitt BM, O’Byrne JM. Design, implementation and prospective evaluation of a new interactive musculoskeletal module for medical students in Ireland. Ir J Med Sci. 2013;182(2):191–9. 10.1007/s11845-012-0855-0. Epub 2012 Oct 6. PMID: 23054476. 23054476 10.1007/s11845-012-0855-0

[17] Cai W, You M, Li J, Li Q, Wang D, Wang H. Application of immersive contextualization based-learning teaching mode in the orthopaedic musculoskeletal disorder module of clinical medicine education. BMC Med Educ. 2023;23(1):906. 10.1186/s12909-023-04831-y. PMID: 38031076; PMCID: PMC10687819. 38031076 10.1186/s12909-023-04831-y PMC10687819

